# Connectivity of Coral Reefs Along the Kuroshio Current Calls for Transboundary Conservation Strategies

**DOI:** 10.1002/ece3.72203

**Published:** 2025-09-20

**Authors:** Naoki Saito, Akira Iguchi

**Affiliations:** ^1^ Integrated Research Center for Nature Positive Technology National Institute of Advanced Industrial Science and Technology (AIST) Tsukuba Japan; ^2^ Geological Survey of Japan National Institute of Advanced Industrial Science and Technology (AIST) Tsukuba Japan

**Keywords:** coral reef, ecological connectivity, Kuroshio current, larval dispersal, Northwest Pacific, transboundary conservation

## Abstract

Environmental conservation becomes more effective when ecological connectivity between patchy habitats is maintained. The coral reef ecosystems of the Yaeyama and Miyako Islands (YAE) in Japan are highly biodiverse and culturally significant but have deteriorated over recent decades. Although coral larvae are expected to be supplied to YAE via the Kuroshio Current from regions outside Japan, previous population genetic and biophysical studies have focused exclusively on connectivity among Japanese coral populations. In this study, we conducted biophysical modelling of 30 years of larval dispersal across the Northwest Pacific using Lagrangian particle tracking, aiming to identify major sources of coral larvae to YAE. The model showed that 86% of virtual larvae reaching YAE represented self‐recruitment. Of the externally sourced virtual larvae, ~70% came from the Philippines, ~20% from Taiwan and only a few percent from Japan. The Kuroshio Current acted as a corridor facilitating dispersal from the northeast Philippines and eastern Taiwan, while simultaneously serving as a barrier to retrograde or transverse dispersal from northern Taiwan and Japan. These findings suggest that most externally supplied larvae to YAE originate from regions outside Japan, upstream of the Kuroshio Current. This study highlights that transboundary collaboration is crucial to understanding and maintaining connectivity between coral reef ecosystems along ocean currents.

## Graphical abstract

We conducted biophysical modelling of 30 years of larval dispersal across the Northwest Pacific to identify major sources of coral larvae to the Yaeyama and Miyako Islands (YAE) in Japan. Of the externally sourced larvae reaching YAE, approximately 70% came from the Philippines, around 20% from Taiwan and only a few percent from Japan. Our findings highlight that transboundary collaboration is crucial to understanding and maintaining connectivity between coral reef ecosystems along the Kuroshio Current.

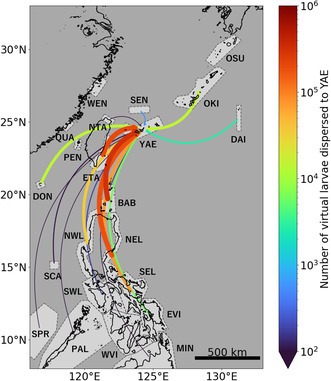

## 1 Introduction

Maintaining ecological connectivity between patchy habitats through the movement of organisms is a priority in environmental conservation (Hilty et al. 2020; Fontoura et al. 2022). Such connectivity enhances the resilience of individual populations to disturbances and supports their long‐term viability (Carlson et al. 2014; Holbrook et al. 2018; Ralls et al. 2018). In terrestrial ecosystems in particular, transboundary conservation efforts have been increasingly recognised as important in recent years to maintain continuous habitats and migration corridors (Kark et al. 2009; Mason et al. 2020). Marine organisms typically exhibit more extensive and higher ecological connectivity than terrestrial organisms (Cowen and Sponaugle 2009; Fontoura et al. 2022). Therefore, effective marine conservation requires an understanding of large‐scale ecological connectivity that spans across national and regional boundaries (Mackelworth 2012; Mazor et al. 2013).

**FIGURE 1:**
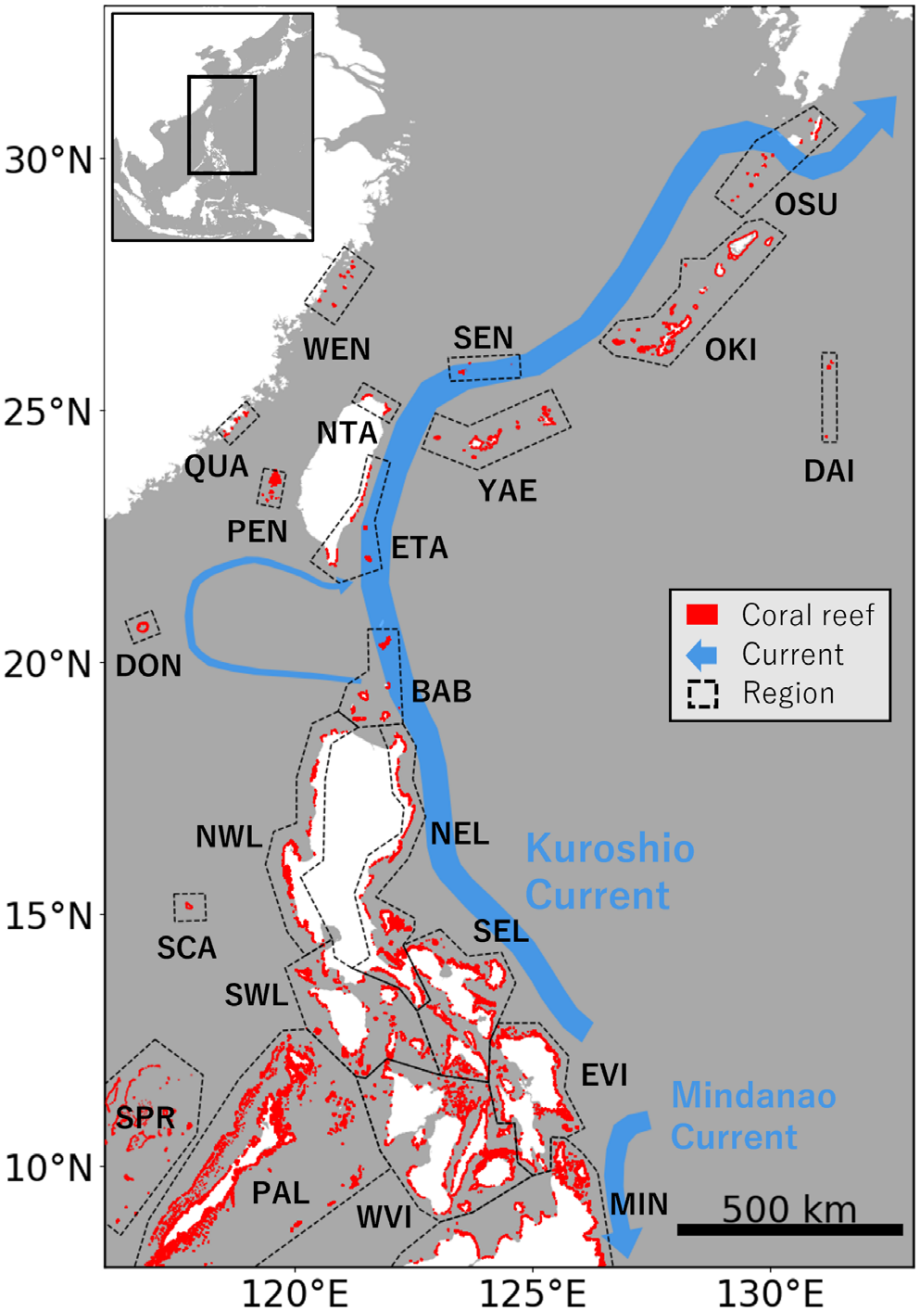
Study area. Red‐shaded areas indicate the distribution of coral reefs from UNEP‐WCMC et al. (2021), and blue arrows indicate the major ocean currents. Black dashed lines delineate the geographical regions of coral reefs as defined in this study, which align with administrative boundaries as much as possible.

The Yaeyama and Miyako Islands (YAE; Figure 1), located in southern Japan, are home to Sekisei Lagoon, the largest coral reef in the country. The reef ecosystems in this region are notably rich in biodiversity on a global scale (Roberts et al. 2002). Furthermore, coral reefs in YAE provide important nature's contributions to people (Díaz et al. 2018) as a foundation for the traditional culture and economy in local communities (Sugimoto et al. 2022). However, as observed in coral reefs worldwide (Hughes et al. 2017), coral populations in YAE have been deteriorating in recent decades (Nakamura 2017; Yasumoto et al. 2025). Damage from mass bleaching events (Hughes et al. 2018; Reimer et al. 2024) has been particularly severe; in 2016, more than 98% of coral colonies of various species in Sekisei Lagoon bleached or were killed (Nakamura 2017). For many coral species, dispersal during the planktonic larval stage is almost the only opportunity to reach other habitats (Cowen and Sponaugle 2009; Randall et al. 2020). The supply of individuals through larval dispersal contributes to the demographic recovery (Holbrook et al. 2018; Hughes et al. 2019) and maintenance of genetic diversity (Carlson et al. 2014; Ralls et al. 2018) of the local populations. For the understanding and conservation of coral reef ecosystems in YAE, the major sources of coral larvae to the area need to be identified.

Although broader transboundary ecological connectivity is likely, previous studies on YAE have largely focused on connectivity within Japan. Previous population genetic studies of reef‐building corals (e.g., Nakajima et al. 2010; Shinzato et al. 2015; Zayasu et al. 2016; Tsuchiya et al. 2022) have shown that YAE acts as a source of gene flow for other Japanese coral reefs and have speculated on downstream larval supply via the nearby Kuroshio Current (Figure 1). However, genetic connectivity between YAE and other coral populations located further upstream of the Kuroshio Current, such as in Taiwan and the Philippines, remains largely unexplored. This can be partly attributed to restrictions on transboundary genetic sampling. The order *Scleractinia*, which constitutes the majority of reef‐building corals, is listed under the Convention on International Trade in Endangered Species of Wild Fauna and Flora (CITES; CITES 2025), and is subject to strict international regulations. Additionally, compliance with the Nagoya Protocol on access to genetic resources and benefit‐sharing (ABS; Secretariat of the Convention on Biological Diversity 2011) adds further complexity. In this context, biophysical modelling of larval dispersal offers a valuable approach to assessing larval sink‐source relationships without the limitations of genetic sampling (Cowen et al. 2006; Fontoura et al. 2022). Nevertheless, previous biophysical modelling for YAE (Uchiyama et al. 2018; Takeda et al. 2021; Kise et al. 2024) has focused exclusively on connectivity between coral reefs within Japan. To identify larval sources to YAE, it is essential to design a broad, transboundary study area that includes coral reefs upstream of the Kuroshio Current.

In this study, we conducted biophysical modelling of larval dispersal among coral reefs across the Northwest Pacific, including Japan, Taiwan, mainland China and the Philippines. The target species was *Acropora digitifera* , a major reef‐building coral in YAE (Nakajima et al. 2010) and widely distributed throughout the Pacific Ocean (Davies et al. 2015). Virtual coral larvae were released from all coral reefs in the study area (Figure 1; UNEP‐WCMC et al. 2021) over a 30‐year period (1994–2023). The model incorporated age‐dependent declining larval survival rates (Nishikawa and Sakai 2005). Modelled dispersal was analysed to identify the major sources of larvae to YAE. The aim of this study was to address a fundamental gap in our understanding of oceanographic connectivity among coral reefs via the Kuroshio Current. This study provides insights into marine conservation strategies based on transboundary connectivity, applicable not only to YAE but also to other coral reef ecosystems.

## 2 Results

**FIGURE 2:**
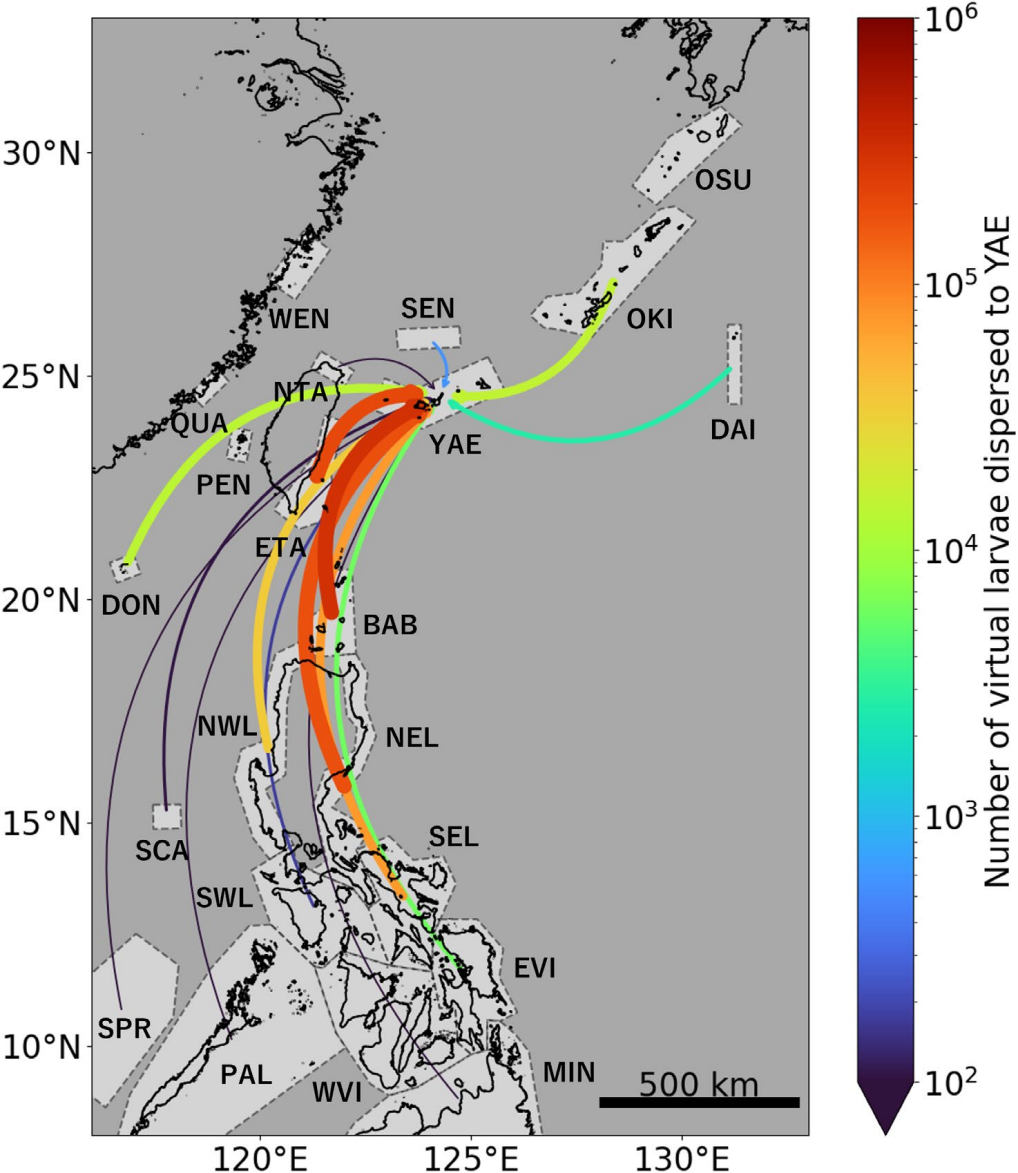
Number of virtual larvae supplied to YAE from each region. Thicker and redder arrows connecting each region to YAE represent a greater number of virtual larvae supplied. Note that the colour bar uses a logarithmic scale. Regions without arrows did not supply any virtual larvae.

**FIGURE 3:**
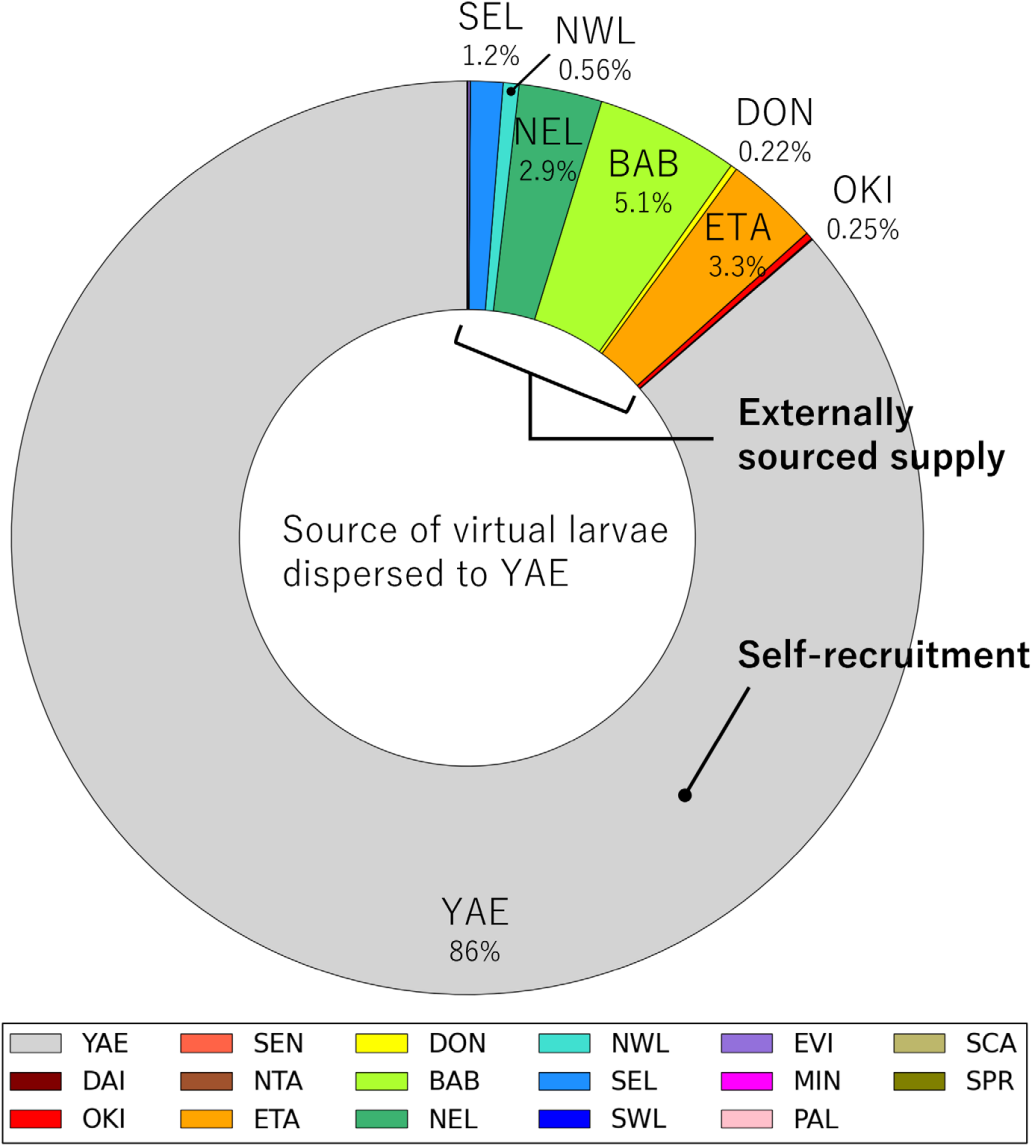
Percentage of virtual larvae from each region among the total dispersed to YAE over a 30‐year simulation. Each region is colour‐coded. Regions not shown in the legend did not supply any virtual larvae to YAE.

As a result of 30 years of dispersal modelling, virtual coral larvae were dispersed to YAE from 15 of the 20 regions targeted (Figure 2). Of the total virtual larvae that dispersed to YAE, 86% represented self‐recruitment from YAE and nearly 10% came from the Philippines (Figure 3). The regions that supplied the most virtual larvae to YAE were the Babuyan and Batanes Islands (BAB), the northernmost islands of the Philippines, which supplied 5.1% of the total. The regions that supplied the second to fifth largest number of virtual larvae were as follows: eastern Taiwan (ETA) contributed 3.3% of the total, northeastern Luzon (NEL) in the Philippines contributed 2.9%, southeastern Luzon (SEL) contributed 1.2% and northwestern Luzon (NWL) contributed 0.56%. The proportion of virtual larvae supplied by Japanese regions other than YAE itself was extremely low, with the highest contribution from the Okinawa and Amami Islands (OKI) at only 0.25%. No virtual larvae were supplied from the Osumi and Tokara Islands (OSU) in Japan, mainland China (WEN and QUA), the Penghu Islands (PEN) and western Visayas (WVI) in the Philippines.

Despite being relatively far away (> 430 km), the Philippines supplied a large number of virtual larvae to YAE. This was partly because the Philippines has the world's third‐largest coral reef area (UNEP‐WCMC et al. 2021) and released many virtual larvae (Figure S1). To exclude the effect of differences in area, we also compared the number of virtual larvae supplied to YAE per unit area of coral reef (0.1° × 0.1°; Figure S2). In this case, as observed in the total supply, the highest supply was from BAB (*N* = 460) and the second highest supply was from ETA (*N* = 251). BAB and ETA supplied over three times as many virtual larvae per unit area as the other regions. The regions ranked third to fifth in per‐unit‐area supply were the Dongsha Islands (DON; *N* = 82), NEL (*N* = 76) and NWL (*N* = 24). Therefore, regardless of the differences in coral reef area, BAB and ETA were strongly connected to YAE through dispersal.

**FIGURE 4:**
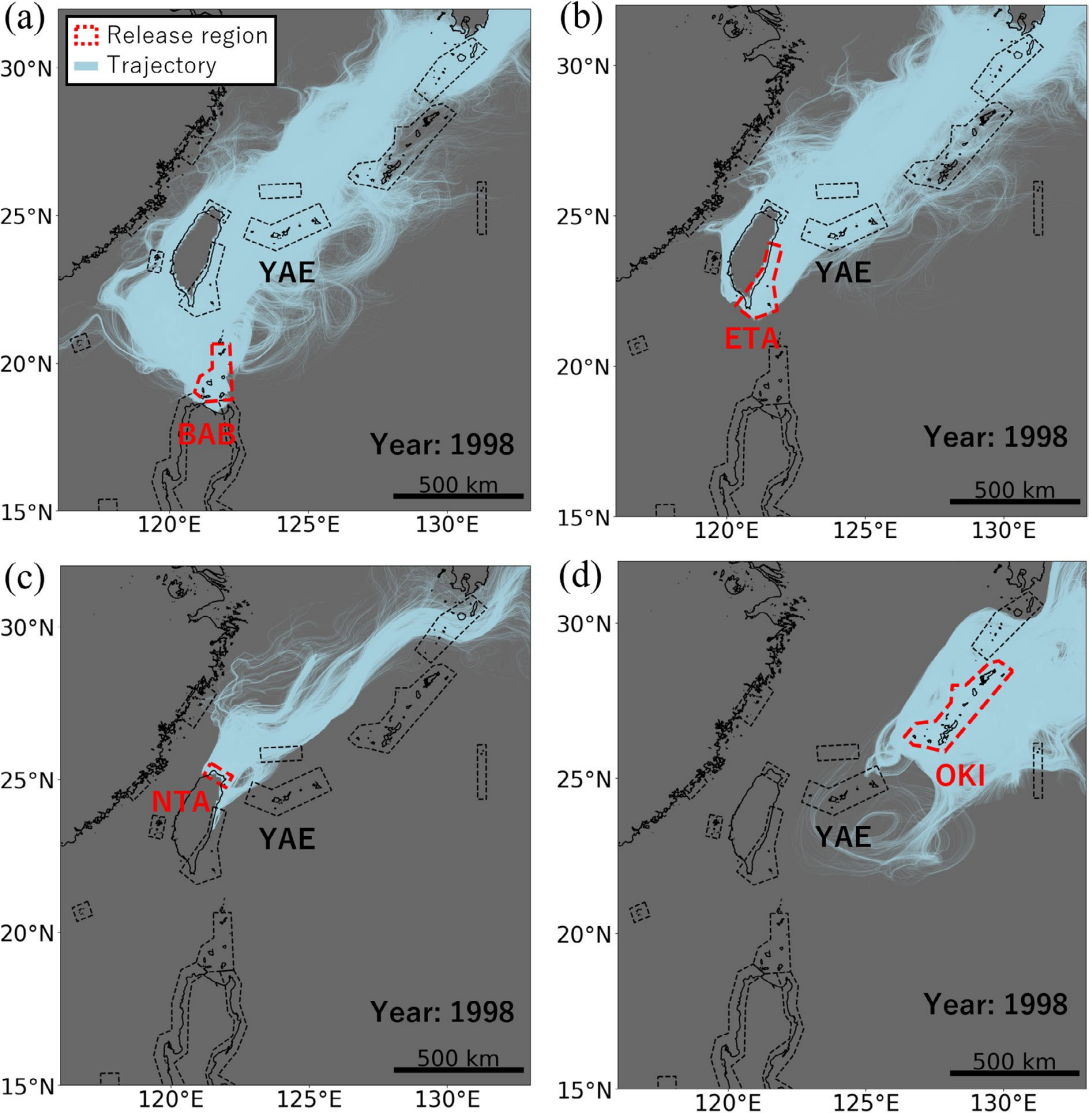
Dispersion trajectories of virtual larvae in 1998. Each panel shows the trajectories of virtual larvae released from (a) BAB, (b) ETA, (c) NTA, and (d) OKI. Light blue lines indicate the dispersion trajectories, and red dotted lines indicate the release regions. For clarity, only the results from 1998 are shown out of the 30 years simulated.

The high larval supply from certain regions to YAE was primarily driven by their locations upstream of the Kuroshio Current. Many virtual larvae from the northeastern Philippines and ETA were transported downstream to the vicinity of YAE along the Kuroshio Current (Figure 4). On the other hand, the Kuroshio Current also acted as a barrier that prevented virtual larvae from dispersing in the opposite direction or crossing the current. Although northern Taiwan (NTA) and the Senkaku Islands (SEN) are adjacent to YAE (> 110 km), the amount of virtual larval supply to YAE from these regions was extremely small, accounting for 0.00034% and 0.0093% of the total, respectively. This was because the Kuroshio Current, passing between NTA/SEN and YAE, prevented the virtual larvae from dispersing across it (Figures 4 and S3). In addition, virtual larvae from downstream regions of Japan, such as OKI and OSU, were prevented from dispersing upstream to YAE by the Kuroshio Current (Figures 4 and S3). DON supplied relatively large numbers of virtual larvae to YAE (Figure 2) despite being distant from the Kuroshio Current (> 450 km). This can be interpreted as virtual larvae from DON being transported to the Kuroshio Current area by the loop current south of Taiwan (Li et al. 1998; Figures S3 and S4).

**FIGURE 5:**
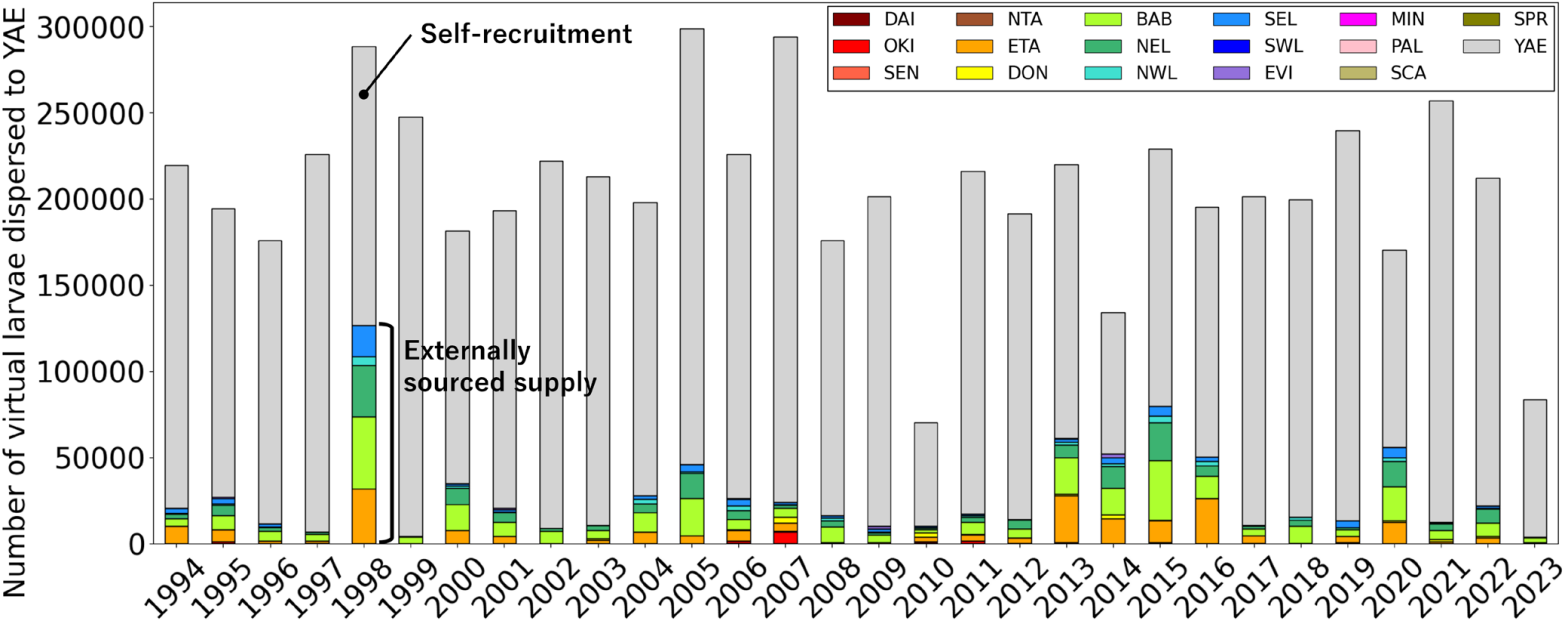
Percentage of virtual larvae from each region among the total dispersed to YAE for each year. Each region is colour‐coded. Regions not shown in the legend did not supply any virtual larvae to YAE.

The number of virtual larvae supplied to YAE, excluding self‐recruitment, varied by up to 32 times from year to year (Figure 5). The supply was particularly high in 1998 (*N* = 126,699) and 2015 (*N* = 79,647), with more than four times the median supply for each year (*N* = 18,932 ± 11,061; median ± interquartile deviation). The lowest supply was observed in 2023 (*N* = 3959). There was a trend for the supply from BAB, ETA and NEL to be particularly high in most years. However, there were also years when this was not the case; for example, in 2007, OKI supplied the largest number of virtual larvae. When including self‐recruitment, the total number of virtual larvae dispersed to YAE was remarkably low in 2010 (*N* = 70,145) and 2023 (*N* = 83,630), and only these years were below half of the annual median (*N* = 206,842 ± 17,116).

## 3 Discussion

This study modelled 30 years of coral larvae dispersal in the Northwest Pacific (Figure 2) and identified major sources of larvae to YAE in Japan. Of the virtual larvae reaching YAE, 86% represented self‐recruitment from YAE (Figure 3). Among externally sourced virtual larvae supplied to YAE, approximately 70% originated from the Philippines, approximately 20% from Taiwan and only a few percent from Japan (Figure 3). The Kuroshio Current acted as a corridor facilitating dispersal from the northeast Philippines and ETA, while simultaneously serving as a barrier to retrograde or transverse dispersal from NTA and Japan (Figure 4). These results suggest that coral reefs outside Japan, located upstream of the Kuroshio Current, supply far more larvae to YAE than those within Japan.

Modelled dispersal (Figure 2) suggests that larval supply from the Philippines and Taiwan may play a critical role in supporting the health and resilience of coral populations in YAE. The finding that most virtual larvae reaching YAE originated from self‐recruitment (Figure 3) reinforces the widely recognised importance of local retention in coral population dynamics and the need to conserve each local population (Mumby and Steneck 2008; Gilmour et al. 2013; Shinzato et al. 2015). However, relying solely on self‐recruitment is unlikely to ensure stable population maintenance (Holbrook et al. 2018; Hughes et al. 2019). Coral populations in YAE are exposed to both global and local stressors, including mass bleaching events (Nakamura 2017), outbreaks of crown‐of‐thorns starfish (Yasuda 2018) and nutrient loading from land (Yasumoto et al. 2025). These disturbances not only reduce local population sizes but also impair their reproductive capacity (Cantin et al. 2007; Johnston et al. 2020). Declines in population density accelerate the loss of genetic diversity through genetic drift, bottlenecks, directional selection and inbreeding (Hernández‐Agreda et al. 2024). Larval supply from distant regions helps prevent such declines in genetic diversity and maintain the adaptive potential to environmental change (Carlson et al. 2014; Ralls et al. 2018). Thus, larval dispersal from the Philippines and Taiwan is likely to contribute both demographically and genetically to the long‐term persistence of coral populations in YAE.

The connectivity between coral reefs shown in this study is also supported by previous biophysical and population genetic studies. The majority (86%) of virtual larvae reaching the YAE were the result of self‐recruitment (Figures 3 and S3), which is consistent with previous biophysical modelling indicating typical larval dispersal distances on coral reefs to be around 100–150 km (Cowen et al. 2006; Treml et al. 2012). Previous population genetic analyses have demonstrated strong genetic connectivity between coastal populations of starfish in Japan and the Philippines (Yasuda et al. 2009; Nakajima et al. 2024) and of gastropods in Japan and eastern Taiwan (Yamazaki et al. 2017). Additionally, gene flow from the northeastern Philippines to Japan has been suggested for seagrasses (Nakajima et al. 2014). In contrast, genetic differentiation has been observed between mudskipper populations in mainland China and Japan (Corush et al. 2022). These population structures are consistent with the Kuroshio Current acting both as a corridor and a barrier to dispersal, as shown in this study (Figure 4). However, differences in dispersal capacity and life history among species make direct comparisons of genetic connectivity challenging (Selkoe et al. 2014; Davies et al. 2015). Broad‐scale population genetic analyses of reef‐building corals covering Japan, Taiwan and the Philippines are therefore still essential to validate the modelled dispersal patterns and to deepen our understanding of ecological connectivity in the Kuroshio Current region.

The supply of virtual larvae to YAE was highly variable, with annual fluctuations of up to 32‐fold (Figure 5). Peak virtual larval supply from external sources occurred in 1998 and 2015, while total virtual larval input, including self‐recruitment, was particularly low in 2010 and 2023. These fluctuations coincide with strong El Niño–Southern Oscillation (ENSO) events (Hughes et al. 2018; Reimer et al. 2024). ENSO weakens the subtropical Kuroshio Current and alters associated eddies, with its effects exhibiting complex interannual variability (Hu et al. 2015; Qiao et al. 2023). This variability in ocean currents could affect the supply of virtual larvae to YAE in this study (Figure S5), as reported for eel larval dispersal in the Northwest Pacific (Kimura et al. 2001; Kim et al. 2007). Interestingly, these strong ENSO events are also known to link with the past four periods of global coral bleaching (Hughes et al. 2018; Reimer et al. 2024). Thus, ENSO‐driven changes in oceanographic connectivity between coral reefs may have influenced the recovery of coral populations after mass bleaching. This may have been the case in YAE, particularly following the 1998 and 2016 mass bleaching events (Nakamura 2017).

Limited data on larval ecology constrain the interpretation of this study's findings. In this study, we calculated 6 million patterns of larval dispersal annually, but in reality, corals may release billions of eggs and sperm per hectare (Randall et al. 2020). Furthermore, while the model assumed an equal number of virtual larvae per unit area released from each coral reef, actual reproductive capacity likely varies across regions (Hughes et al. 2019). In particular, reproductive capacity may be reduced in regions or periods affected by disturbances (Cantin et al. 2007; Johnston et al. 2020). The model incorporated a decline in larval survival with age, based on laboratory observations (Nishikawa and Sakai 2005). However, factors such as predation (Penin et al. 2011) and unfavourable environmental conditions (Randall et al. 2020; Yasumoto et al. 2025) may further lower survival rates in the field. Although empirical data remain limited (Randall et al. 2020), incorporating such ecological processes into future models is essential for simulating more realistic coral population dynamics. The results of this study should be interpreted as a quantification of oceanographic connectivity between coral reefs, rather than precise predictions of the number of coral larvae supplied.

Our findings suggest that transboundary connectivity via ocean currents is an important consideration for the conservation of YAE and other coral reef ecosystems. In planning conservation efforts aimed at the ecological connectivity of YAE, the relatively substantial larval supply from the Philippines and Taiwan (Figure 3) should be considered alongside that from within Japan. Transboundary biological surveys and conservation strategies that take into account connectivity along the Kuroshio Current (Figure 4) can help elucidate and preserve ecological networks (Hilty et al. 2020; Fontoura et al. 2022) among coral reefs in the Northwest Pacific. Such transboundary connectivity of coral populations has also been identified in other regions, including Micronesia (Davies et al. 2015) and East Africa (van der Ven et al. 2016) through population genetic analyses, and in the western tropical Pacific (Kool et al. 2011; Thompson et al. 2018) through biophysical modelling. However, approximately 70% of the world's coral reefs that function as key sources or sinks for larval dispersal remain unprotected (Fontoura et al. 2022). This study underscores that international cooperation to understand and maintain ecological connectivity along ocean currents is key to the effective conservation of coral reef ecosystems.

## 4 Materials and Methods

### 4.1 Study Area

Biophysical modelling was performed for all warm‐water coral reefs located within the area spanning 116° E–133° E and 8° N–33° N (Figure 1). This area covers the latitudinal range from the eastern part of Luzon Island, which is the southernmost extent of the Kuroshio Current (Figure S4), to OSU, the northern limit of coral reef distribution in the western Pacific (UNEP‐WCMC et al. 2021). Longitudinally, the area extends from the loop current off southern Taiwan in the west, a part of the Kuroshio Current that flows into the South China Sea (Li et al. 1998), to OSU in the east.

Coral reef distribution data were sourced from the dataset by UNEP‐WCMC et al. (2021). This dataset, based primarily on satellite imagery and integrating a wide range of sources, is one of the most comprehensive global datasets available (UNEP‐WCMC et al. 2021). For the purposes of this study, coral reefs were classified into twenty geographical regions (Figure 1), delineated according to administrative boundaries wherever possible.

### 4.2 Larval Ecology Settings

The species targeted in the biophysical modelling was *Acropora digitifera* , a reef‐building coral that releases buoyant sperm and eggs into water for fertilisation, forming planula larvae (Nishikawa and Sakai 2005). Given the wide distribution of *A. digitifera* in the Pacific Ocean (Nakajima et al. 2010; Davies et al. 2015), it was assumed that larvae were released from all coral reefs within the study area.

Based on previous laboratory experiments (Nishikawa and Sakai 2005) that recorded the survival rates of *A. digitifera* larvae at different ages, the following survival rates were incorporated into the model: 100% survival rate for 0–4 days after spawning, 55% for 4–10 days, 40% for 10–20 days, 30% for 20–30 days, 15% for 30–40 days and 10% for 40–50 days. Dispersal calculations for all virtual larvae ended 50 days after spawning. Following observations from the same laboratory experiments (Nishikawa and Sakai 2005), the pre‐competency period, which is the time from spawning until larvae mature and become capable of settlement, was set to be 4 days.

The timing of coral mass spawning is related to factors such as rising sea temperatures (Keith et al. 2016). Based on observational data of spawning periods for *Acropora* spp. collected in the Pacific Ocean (Keith et al. 2016), different spawning periods were assigned according to latitude as follows: spawning was assumed to begin on 15 May for latitudes above 25° N, 15 April for 20° N–25° N and 15 March for latitudes below 20° N, with the spawning period lasting 60 days from the start date.

### 4.3 Larval Dispersal Simulation

We conducted larval dispersal simulations using Lagrangian particle tracking analysis to release virtual larvae from coral reefs. The analysis was performed using the Python package ‘Parcels’ (Delandmeter and van Sebille 2019). The dispersal trajectories of virtual larvae were calculated using the following equation:

(1) $$X\left(t+∆t\right)=X\left(t\right)+\int_{t}^{t+∆t}v\left(x,\tau \right)\mathrm{d\tau}$$

where X is the position of a virtual larva, *t* is time and $v\left(x,\tau \right)$ is the linearly interpolated horizontal velocity at that position. As in previous modelling studies (Uchiyama et al. 2018; Takeda et al. 2021; Kise et al. 2024), larvae were assumed to be passively transported by horizontal currents, and vertical migration was not included to reduce model complexity. The dispersal depth was set to be 0 m.

Flow velocity data were obtained from the ocean model JCOPE‐FGO (Kido et al. 2022), which has a horizontal resolution of 0.1° and a vertical resolution of 44 layers in the σ‐coordinate system. Model outputs are provided at daily intervals. The accuracy of JCOPE‐FGO is enhanced by assimilating observational datasets, including satellite‐derived sea level and surface water temperature, as well as in situ water temperature and salinity profiles. JCOPE‐FGO has been validated to accurately reproduce observed surface currents worldwide, including the Kuroshio Current (Kido et al. 2022).

Virtual larvae were released from 1123 sites (Figure S1) located within the coral reefs in the study area, spaced at intervals of 0.1° to match the horizontal resolution of JCOPE‐FGO. The simulation period covered 30 years, from 1994 to 2023. Virtual larvae were released daily during the spawning season, which varies by latitude (as described above). In each release, 100 virtual larvae were randomly placed within a 0.1° × 0.1° area. This resulted in 6000 virtual larvae being released per site during the annual 60‐day spawning season, totalling 180,000 per site over 30 years. Across a total of 1123 sites, more than 6 million virtual larvae were released annually, amounting to more than 200 million over 30 years. Based on age‐specific survival rates (as described above), a random subset of virtual larvae released from each site was assumed to have died, and their dispersal calculations were accordingly terminated. The calculation domain spanned 113° E–136° E and 5° N–36° N, including a buffer zone extending 3° or more beyond the target coral reefs. The dispersal calculation for a virtual larva was completed once it exited this domain. The calculations were performed through parallel processing for each year using the ‘ProcessPoolExecutor’ class in the Python module ‘concurrent.futures’ (https://docs.python.org/3/library/concurrent.futures.html, accessed 9 July 2025). The CPU used was an Intel Core i9‐13900K (3.00 GHz).

In the analysis of the simulation results, we first calculated the number of virtual larvae released from site i and passing through the 0.1° × 0.1° area centred on site j ($n_{i\to j}$). Only passages occurring after the four‐day pre‐competency period (as described above) were included in the analysis. Next, the total number of virtual larvae released from region A and reaching YAE ($N_{A\to \mathrm{YAE}}$) was calculated as follows:

(2) $$N_{A\to \mathrm{YAE}}=\sum_{i\in S_{A}}\sum_{j\in S_{\mathrm{YAE}}}\left(n_{i\to j}\right)$$

where $S_{A}$ is the set of all release sites within region A. This value ($N_{A\to \mathrm{YAE}}$) was calculated for each year of release and then summed over the 30‐year simulation period.

## Author Contributions

**Naoki Saito:** conceptualization (lead), formal analysis (lead), writing – original draft (lead). **Akira Iguchi:** writing – review and editing (lead).

## Conflicts of Interest

The authors declare no conflicts of interest.

## Supporting information


**Data S1:** ece372203‐sup‐0001‐Supinfo.pdf.

## Data Availability

The data that support the findings of this study are openly available in Zenodo at https://zenodo.org/records/15487733.
