# Supplementary material for: Connectivity of Coral Reefs Along the Kuroshio Current Calls for Transboundary Conservation Strategies
Source: Ecol Evol. 2025 Sep 20;15(9):e72203. doi: 10.1002/ece3.72203 (PMC12449672; doi:10.1002/ece3.72203)
Supplement: Supplementary file 1 — Data S1: ece372203‐sup‐0001‐Supinfo.pdf. [file ECE3-15-e72203-s001.pdf]

Supporting information for

Connectivity of coral reefs along the Kuroshio Current calls for transboundary conservation strategies

Figures

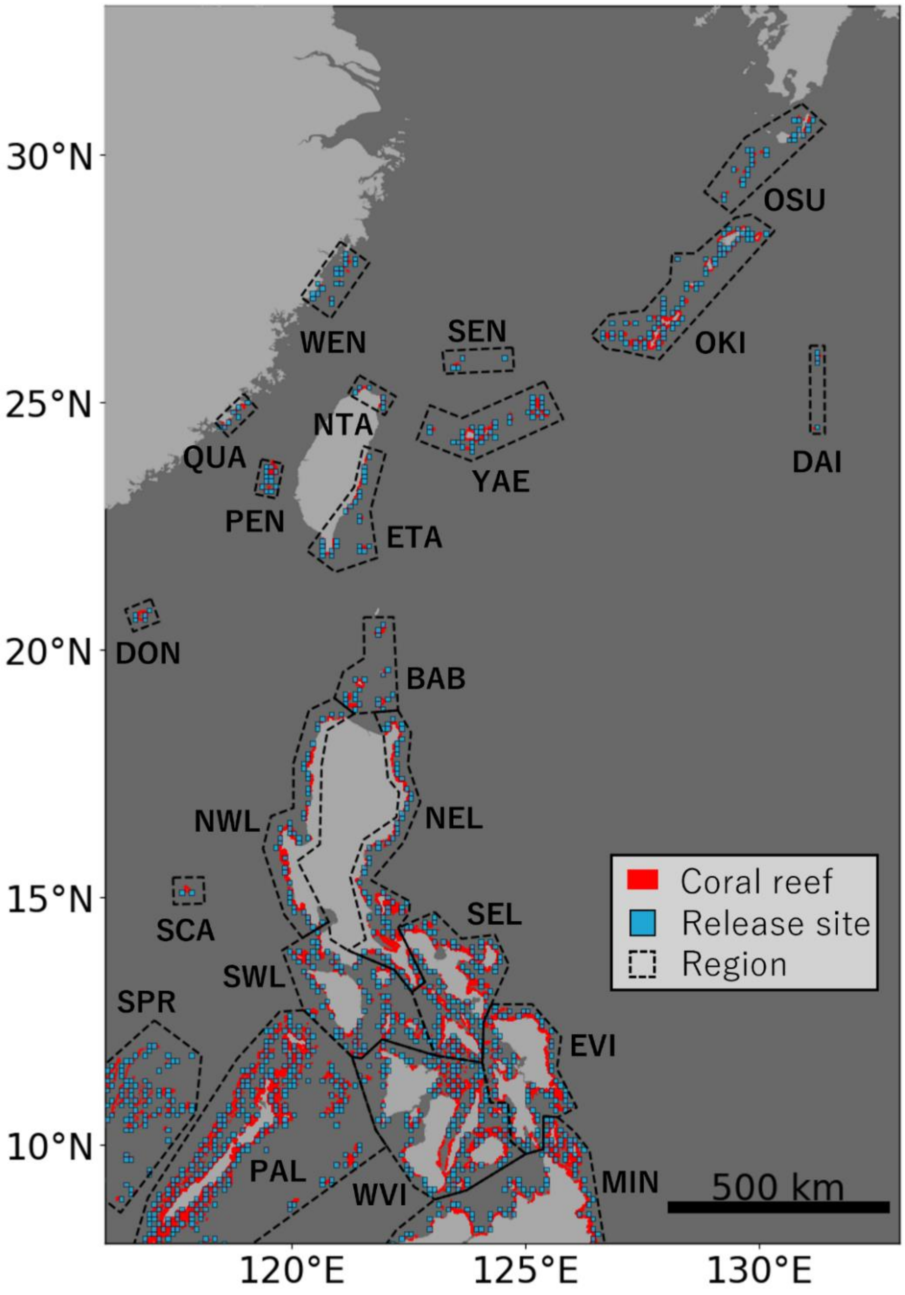

Fig. S1. Virtual larvae release sites. Blue squares indicate virtual larvae release sites, and red-shaded areas indicate the distribution of coral reefs from UNEP-WCMC et al. (2021). Black dashed lines delineate the geographical regions of coral reefs as defined in this study, which align with administrative boundaries as much as possible.

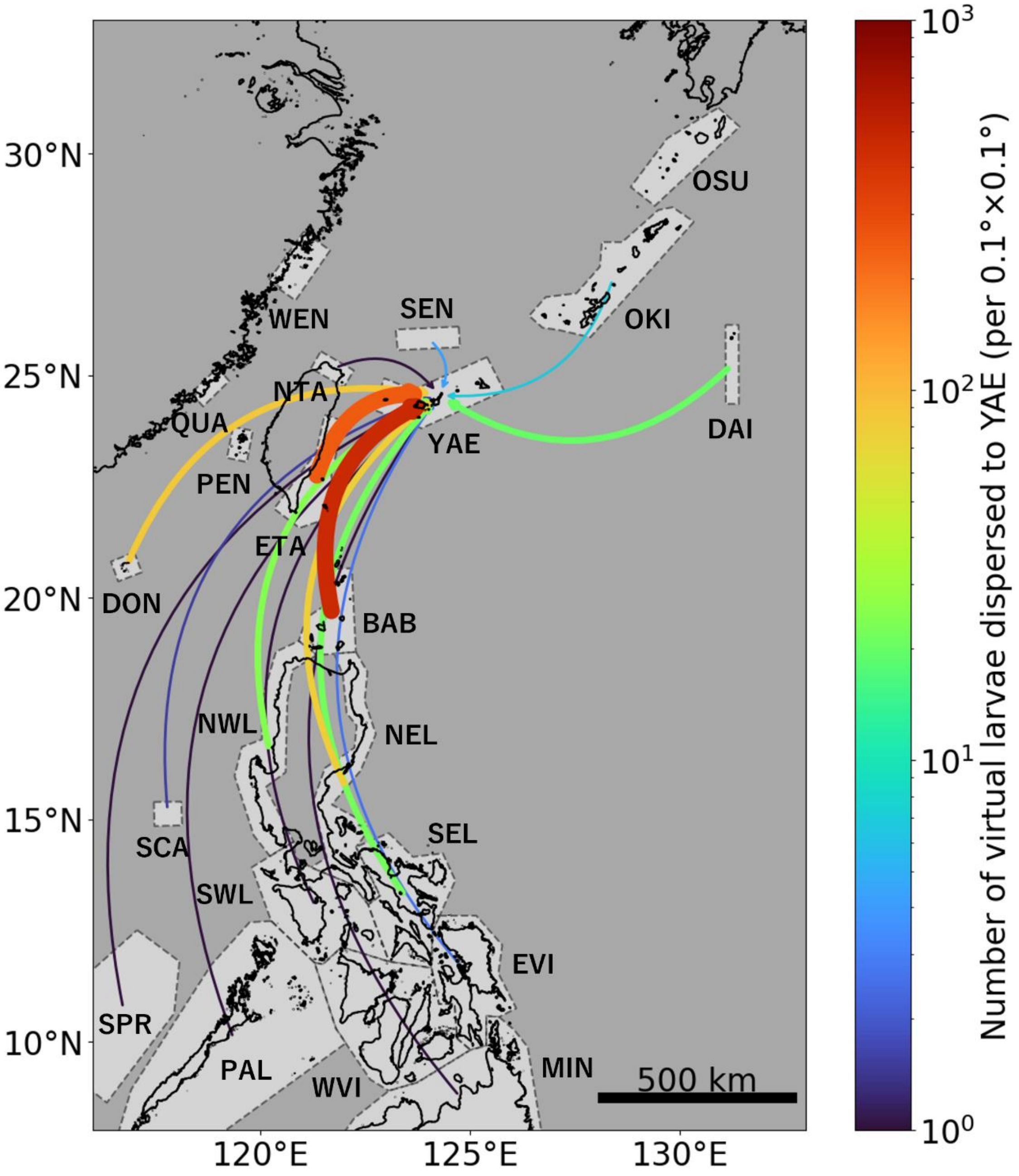

Fig. S2. Number of virtual larvae supplied to YAE from each region per unit area of coral reef (0.1° × 0.1°). Thicker and redder arrows connecting each region to YAE represent a greater number of virtual larvae supplied. Note that the colour bar uses a logarithmic scale. Regions without arrows did not supply any virtual larvae.

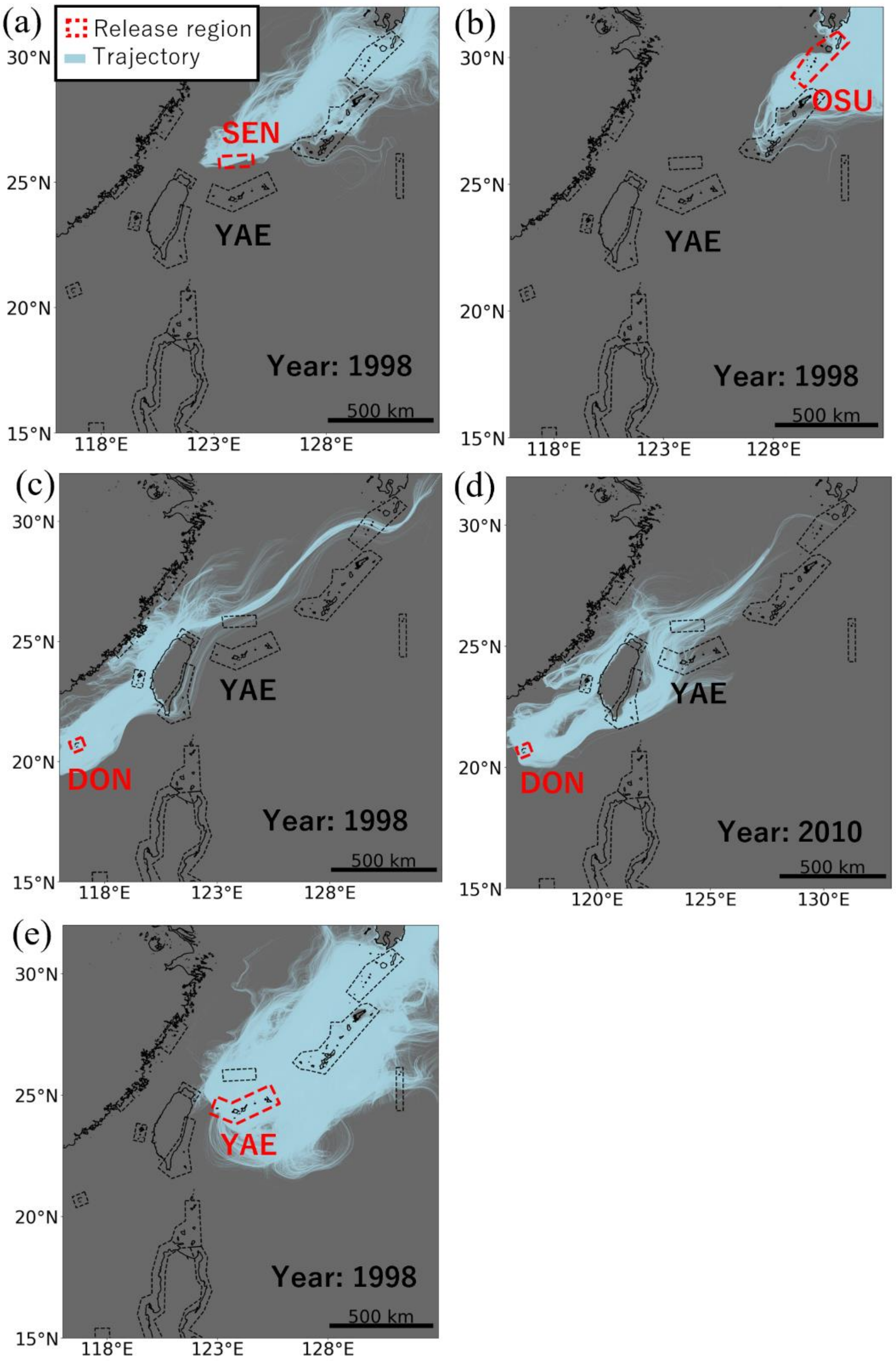

Fig. S3. Dispersion trajectories of virtual larvae. Each panel shows the trajectories of virtual larvae released from (a) SEN, (b) OSU, (c, d) DON, and (e) YAE. (a–c) and (e) show dispersion in 1998. (d) shows dispersion in 2010, when a relatively large number of virtual larvae dispersed from DON to YAE. Light blue lines indicate the dispersion trajectories, and red dotted lines indicate the release regions. For clarity, each panel shows the results from only one year out of the 30 years simulated.

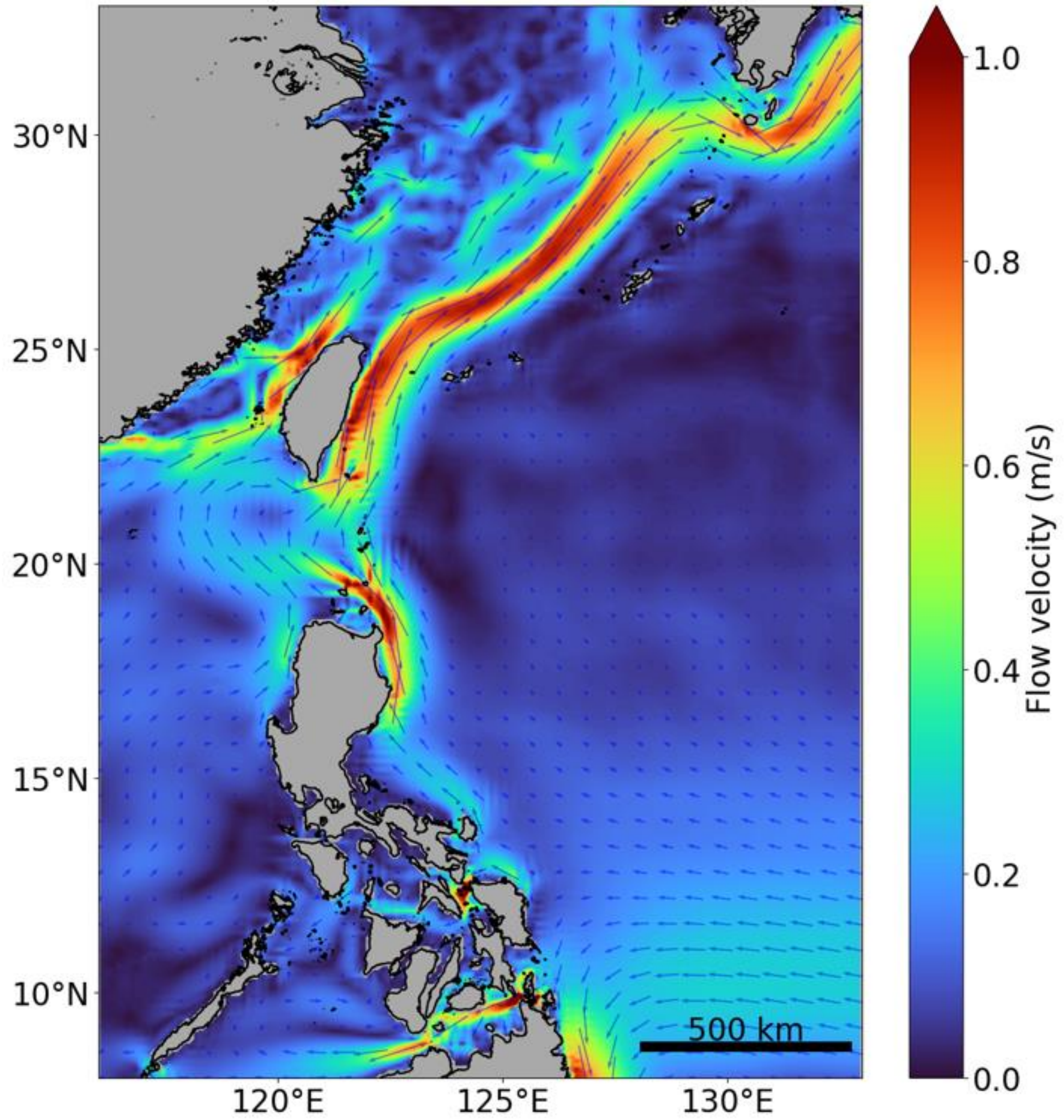

Fig. S4. Mean ocean flow from March to September, averaged over the period 1994–2023. March to September corresponds to the season of virtual larval dispersal. Flow velocity data were obtained from the ocean model JCOPE-FGO.

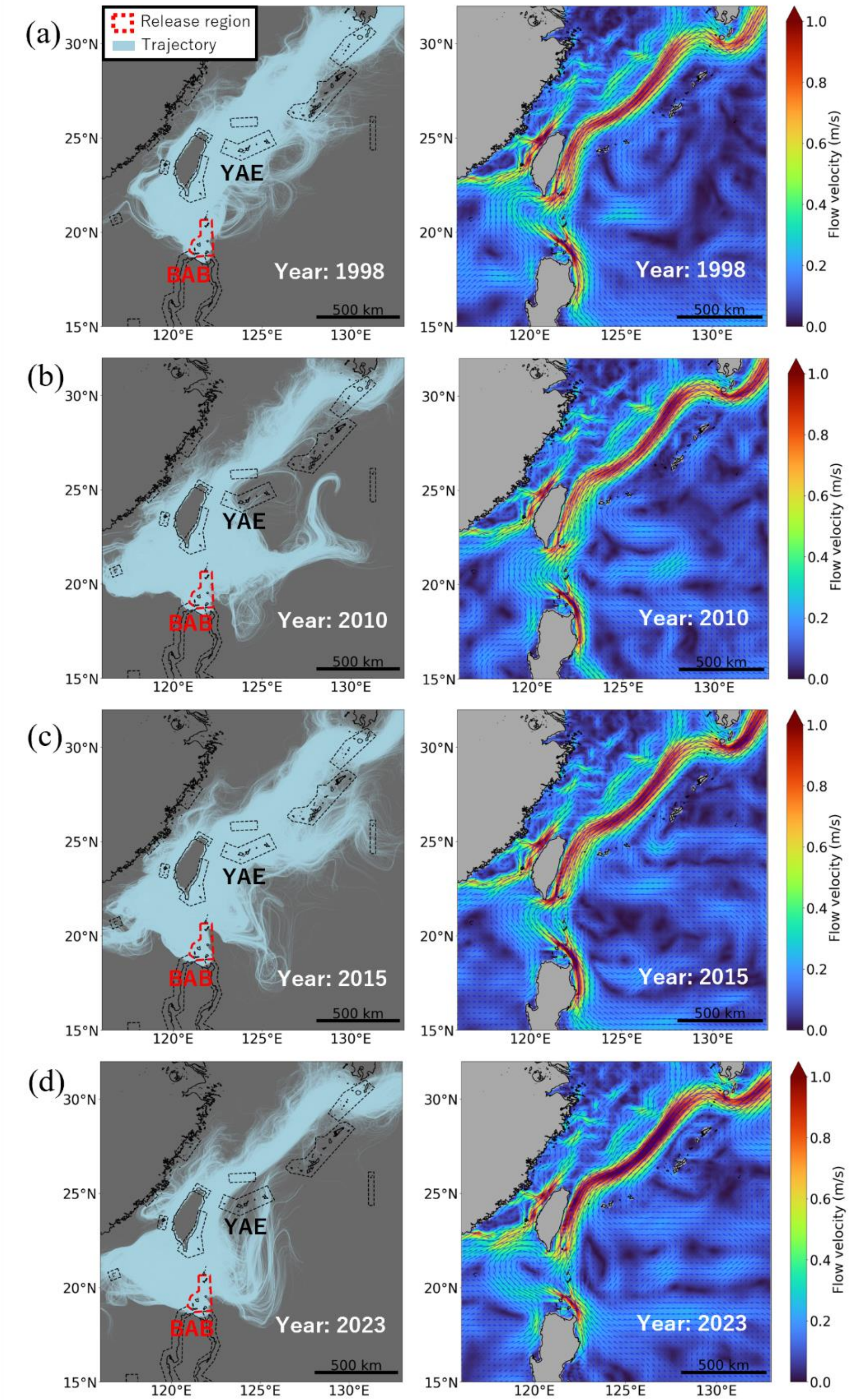

Fig. S5. Dispersal trajectories of virtual larvae released from BAB in each year (left side of each panel) and mean ocean flow from March to September of the same year (right side of each panel). Results are shown for (a) 1998, (b) 2010, (c) 2016, and (d) 2023. Light blue lines indicate the dispersion trajectories, and red dotted lines indicate the release regions. March to September corresponds to the season of virtual larval dispersal. Flow velocity data were obtained from the ocean model JCOPE-FGO.

Reference

UNEP-WCMC, WorldFish Centre, WRI, & TNC. *Global Distribution of Warm-Water Coral Reefs, Compiled from Multiple Sources Including the Millennium Coral Reef Mapping Project. Version 4.1*. http://data.unep-wcmc.org/datasets/1 (2021).
